# Ecology and Distribution of Thaumarchaea in the Deep Hypolimnion of Lake Maggiore

**DOI:** 10.1155/2015/590434

**Published:** 2015-08-25

**Authors:** Manuela Coci, Nina Odermatt, Michaela M. Salcher, Jakob Pernthaler, Gianluca Corno

**Affiliations:** ^1^Microbial Ecology Group, CNR-Institute of Ecosystem Study, Largo Tonolli 50, 28922 Verbania, Italy; ^2^Microb&Co, Association for Microbial Ecology, Viale XX Settembre 45, 95128 Catania, Italy; ^3^Limnological Station, Institute of Plant Biology, University of Zurich, Seestrasse 187, 8802 Kilchberg, Switzerland; ^4^Institute of Hydrobiology, Biology Centre CAS, Na Sádkách 7, 370 05 České Budějovice, Czech Republic

## Abstract

Ammonia-oxidizing Archaea (AOA) play an important role in the oxidation of ammonia in terrestrial, marine, and geothermal habitats, as confirmed by a number of studies specifically focused on those environments. Much less is known about the ecological role of AOA in freshwaters. In order to reach a high resolution at the Thaumarchaea community level, the probe MGI-535 was specifically designed for this study and applied to fluorescence *in situ* hybridization and catalyzed reporter deposition (CARD-FISH) analysis. We then applied it to a fine analysis of diversity and relative abundance of AOA in the deepest layers of the oligotrophic Lake Maggiore, confirming previous published results of AOA presence, but showing differences in abundance and distribution within the water column without significant seasonal trends with respect to Bacteria. Furthermore, phylogenetic analysis of AOA clone libraries from deep lake water and from a lake tributary, River Maggia, suggested the riverine origin of AOA of the deep hypolimnion of the lake.

## 1. Introduction

The recently described deep-branching phylum in the archaeal domain, Thaumarchaea (formerly mesophilic Crenarchaea), represents one of the most abundant groups of Archaea, being found in a variety of environments including soils, oceans, and freshwaters [1, 2]. They significantly contribute to the global nitrogen and carbon cycle through chemolithoautotrophic oxidation of reduced nitrogen compounds. The phylum includes ammonia-oxidizing Archaea (AOA).

AOA potentially compete with ammonia-oxidizing Bacteria (AOB) because they share the same substrate and the same ecological niche. A better understanding of their interactions and relative contributions to the global nitrogen cycle is needed. Depending on the substrates (soils or waters), several factors can influence AOA success: pH, temperature, ammonium, oxygen availability, trace element concentration, and light intensity [3]. The results from previous studies are rather contrasting: AOA have been found in significant proportions in open oceans (up to 20% of all prokaryotic community [4]) and in soils, while other studies found relative dominance of AOB in terrestrial environments [5]. Such kinds of measurements and of detailed observations are still missing for freshwaters.

Studies on the open ocean water column suggested that AOA are likely to be more abundant in the deeper layers whereas AOB are ecologically successful in the upper ones [6]. Reasons for this spatial niche separation could be the high specific affinity of AOA to ammonia, making environments with low NH_4_^+^ concentrations preferential habitats for AOA [7], the dependency of AOA on the availability of trace metals for the high number of copper-containing enzymes involved in the archaeal ammonia oxidation, some of which are unique for Archaea [8, 9], and the sensitivity of Archaea to UV radiation [10].

Only in the last 15 years have Archaea been recognized as a group of potential interest in lakes and rivers, and only in the last 5 years have freshwater Thaumarchaea been considered as important players for ammonia oxidization. Early studies on their ecology focused on very specific aquatic freshwater environments (e.g., biofilters [11], sulfurous karstic Lake Vilar [12], arctic saline lakes [13], and deep-glacial cirque Lake Redon [14]) observing significant differences in distribution and overall richness through the seasons. A study on the oligomictic Lake Lucerne [15] revealed limited seasonality patterns (in general related to changes in climatic conditions) and parallel temporal variations of AOA and AOB, indicating limited competition for ammonium, or broader resource spectra for AOA. Finally a more recent study on abundance and diversity of AOA and AOB in the Great Lakes system revealed AOA niche differentiation based on sampling location and thereby trophic states of the lakes [16]. AOA had been described also in rivers [17]. AOA have been already found in a pilot study in Lake Maggiore, where their abundance, as well as their contribution to dark inorganic carbon assimilation, significantly increased in the hypolimnion [18, 19], however, without providing important phylogenetic information.

Phylogenetic clades within Thaumarchaea are mostly named on the base of the habitat where the sequences were first collected [2]: Marine Group I (MGI) Thaumarchaeota (formerly 1.1a Crenarchaea), commonly found in marine and freshwater plankton, Soil Group (formerly 1.1b Crenarchaea), ThAOA/HWCG III (Hot Water Crenarchaeotic Group III from a hot water stream in a gold mine), and SAGMG-1 (South African Gold Mine Group). More recently, these four groups were associated with representative genera:* Nitrosopumilus* (MGI, Thaumarchaea Marine Group I.1a),* Nitrososphaera* (Thaumarchaea Soil Group I.1b),* Nitrosocaldus* cluster (ThAOA/HWCG III), and* Nitrosotalea* (SAGMG-1) [16].

The 16S rRNA phylogeny of Thaumarchaea suggests a rather defined intragroup differentiation between marine and terrestrial clades; the freshwater groups are in some cases closely related to the firsts and in some other cases to the seconds, demonstrating the need of a better resolution of freshwater AOA distribution [20]. This study focused on “Thaumarchaea ammonia oxidizers” in the deep layers of the Lake Maggiore along a year and in the tributary River Maggia, to better resolve the resident populations and to identify potential inputs of allochthonous strains from the catchment area, through a phylogenetic comparison between the different Thaumarchaea OTUs in the lake and the river.

## 2. Material and Methods

Lake Maggiore is located between Italy and Switzerland, at the southern margin of Western Alps, and included in the LTER network (LTER: Long-Term Ecological Research, http://www.ise.cnr.it/lter). It is one of the deepest subalpine lakes with a maximum depth of 372 m and a very large catchment area of about 6600 km^2^. Urban areas surround the Lake, while the drainage basin is characterized by a prevalence of high mountains and a relatively low impact of agricultural activities. After the eutrophication event in the 1960s, Lake Maggiore shifted to a mesotrophic state and recovered in the beginning of the 1990s and since then has been very oligotrophic [21]. Lake Maggiore is currently defined as holo-oligomictic [22]: the full mixing of large water masses only occurred after a very cold winter period (i.e., 1956, 1963, and 1970), while usual winter mixing reaches 100–200 m depth. The deeper layers are fed by cold oxygenated waters from the rivers, as demonstrated in the years 1999, 2000, 2004, 2005, and 2006 [23]. The third largest catchment area of Lake Maggiore is represented by the River Maggia basin (926.10 km^2^) with an inflow 50 m below the lake surface, mediated by the artificial construction of a hydropower plant. Due to their low temperature, Maggia waters quickly sink to about 200 m depth.

### 2.1. Sample Collection

Sampling was conducted twice a month from March to October and once in November and December 2011 (18 dates in total) at the traditional long-term sampling point of Lake Maggiore (Ghiffa pelagic station; 45°57′N, 3°46′W, about 370 m depth) to estimate prokaryotic abundance and community composition and to determine biotic and abiotic parameters. Water samples of 5–10 L were collected with Niskin bottles at 3, 10, 50, 200, and 350 m depth and transported in dark plastic tanks previously rinsed with 0.01% HCl to prevent contaminations. Profiles of pH, temperature, oxygen concentration, chlorophyll* a*, conductivity, and light intensity were determined with the multiparameter probe OCEAN SEVEN 316 (IDRONAUT) with a density of one measurement every 0.5 m. Profiles of reactive phosphorus, ammonium, nitrite, and nitrate were measured at 0, 5, 10, 20, 30, 50, 10, 150, 200, 250, 300, and 350 m depth at the Chemistry Lab of the CNR-Institute of Ecosystem Study. Samples for total and dissolved organic carbon (TOC and DOC) and dissolved inorganic carbon (DIC) were collected at 50, 200, and 350 m during each sampling and measured by an OC Analyzer (Shimadzu Corp.). Water samples (5–10 L) of River Maggia were collected from the river shore in 0.01% HCl rinsed plastic tanks in spring and summer and used to assess the ammonia-oxidizing prokaryotic community.

### 2.2. Microbial Abundance and Community Composition

Total prokaryotic abundance was determined from surface (3 and 10 m) and deep water layers (200 and 350 m): 100 mL of each sample were fixed with formaldehyde (2% final concentration); 1 mL aliquots were stained with DAPI (4,6-diamidino-2-phenylindole), filtered on black polycarbonate filters (0.22 *μ*m pore size; GTTP, Millipore), and enumerated by epifluorescence microscopy (Axioplan, Zeiss). At least 1000 DAPI stained cells per filter were counted. Total prokaryotic abundance was used to calculate total numbers of Thaumarchaea from the hybridization rates of the community composition analysis at 200 and 350 m.

The composition of the prokaryotic community was determined by fluorescence* in situ* hybridization followed by catalysed reporter deposition (CARD-FISH [26]) performed on 8 mL samples from 200 m and 350 m that were filtered on white polycarbonate filters (0.22 *μ*m pore size; GTTP, Millipore) and embedded in 0.1% ultrapure agarose (wt./vol., Sigma-Aldrich). CARD-FISH probes (Table 1) used in this study were EUB I–III (targeting Bacteria [25]), ARC915 (Archaea [24]), and MGI-535 (Thaumarchaea of the Marine Group I, specifically designed for this study; see below). Probe MGI-535 was tested at different formamide concentrations in the hybridization buffer until the highest stringency was obtained at 45%. CARD-FISH was performed as described by Sekar et al. [27] with minor modifications [28]. CARD-FISH analyses for MGI-Thaumarchaea were conducted in triplicate and filters were analysed by fully automated microscopy and image analysis [29]. At least 400 positive CARD-FISH cells and 1000 DAPI cells were evaluated per filter piece.

### 2.3. Analysis of Ammonia-Oxidizing Prokaryotic Communities

The spatial distribution of ammonia-oxidizing Bacteria and Archaea was assessed with PCR-DGGE fingerprinting technique from 50, 200, and 350 m samples of Lake Maggiore and from the River Maggia in April and June and in July and August, respectively. For prokaryotic DNA collection, 2.5 L water samples were filtered on a Sterivex filter unit (0.22 *μ*m pore size, Millipore) further divided into two pieces with a sterile scalpel. DNA was isolated from one half of each filter with the UltraClean Soil DNA Isolation Kit (Mo-Bio), with minor modification from the manufacturer's protocol for maximum yield: 0.5 g of sterilized zirconia beads (0.1 mm diameter) was used instead of the beads supplied [30] and the DNA was eluted in 60 *μ*L of nuclease-free water (Promega). The extracted DNA was quantified by using a high sensitivity Nanophotometer (Implen GmbH).

Polymerase chain reactions (PCRs) were performed with a MyCycler (Bio-Rad Laboratories) thermocycler, with 80–100 ng genomic DNA, GoTaq Green Master Mix (Promega), and different primers sets (Table 2) to target both the 16S rRNA and the ammonia-monooxygenase genes (*amo*A) of ammonia-oxidizing Bacteria and Archaea. Presence of PCR inhibitors in River Maggia samples was excluded by mixing DNA samples from the lake and the river at different ratios and evaluating the degree of contamination of the diverse amplicons.

To assess dynamics of ammonia-oxidizing prokaryotes PCR amplicons were separated by Denaturing Gradient Gel Electrophoresis (DGGE) as described by Muyzer and Smalla [31] using a DCodeTM Universal Mutation Detection System (Bio-Rad Laboratories, Inc.). Electrophoresis was run for 16 h applying the following conditions: 100 V, 60°C, denaturing gradients 35–60% for 16S rRNA and* amo*A genes of AOB, 20–55% for 16S rRNA of AOA, and 25–45% for* amo*A genes of AOA. DGGE gels were stained in SYBR Green (Thermo Fisher Scientific Inc.) for 45 minutes and further visualized by GelDoc XR System (Bio-Rad Laboratories, Inc.). Analysis of band intensity was performed with the software ImageJ [36] where the intensity of every band was first corrected for the corresponding background and the mean intensity for every lane was calculated by summing up band intensities and dividing them by the number of bands. Mean intensity per lane was normalized for all lanes in order to get comparable relative values. In addition, single bands obtained from the different gels (16S rRNA and amoA genes) were prepared for sequencing reactions, with a procedure of excision, amplification with no-GC containing primers, and separation by electrophoresis for 5 hours at the respective denaturing conditions [37].

### 2.4. Phylogenetic Analysis of Thaumarchaea

Three clone libraries of archaeal 16S rRNA genes were produced from a spring and an autumn sample of Lake Maggiore (350 m depth) and a summer sample of River Maggia for phylogenetic comparison. Considering the absence of seasonal trends, we selected DNA samples with a 260/280 ratio as close as possible to 1.8 (generally accepted for pure DNA). A new reverse primer specifically targeting Thaumarchaeota (thaum922r: 5′-TTG TGG TGC TCC CCC GCC-3′) was designed in ARB using the respective tools [38]. Cloning of PCR amplified fragments of approximately 900 bp length (see Table 2 for details on primers and PCR conditions) was carried out using the pGEM-T Vector System I (Promega) following the manufacturer's protocol. Clones with the correct insert were used for plasmid extraction (GenElute HP Five-Minute Plasmid Miniprep Kit, Sigma) and subsequently sequenced with plasmid primers on an ABI 3730 DNA analyzer (Applied Biosystems). Sequences were assembled with DNA Baser software and checked for chimeric structures with pintail (version 1.0), using* Nitrosopumilus maritimus* as subject sequence. 77 contigs of 900 bp length were aligned online with the SINA web aligner [39] and merged into the SILVA SSU reference database (release 119) in ARB [38]. After manual refinements of the alignments, several close relatives of the obtained sequences were selected, and a bootstrapped maximum-likelihood tree (GTR-GAMMA model, 1000 iterations) was calculated on a dedicated webserver [40]. Probe design for MGI-Thaumarchaea was done with the respective tools in ARB following the workflow described in Salcher et al. [41]. The obtained probe was tested* in silico* for hybridization efficiency and mismatch stability with the web-tool mathFISH [42]. Specificity of the newly designed probe and primer was checked with the TestProbe function in SILVA [39]. Operational taxonomic units (OTUs) were calculated based on 98% sequence identity and rarefaction curves were calculated with Chao1 estimator. All sequences have been deposited to GenBank with accession numbers KP866330–KP866404.

### 2.5. Statistical Analyses

Statistical analyses were performed with the software packages R (R Development Core Team 2011) and SPSS (IBM Corp., released 2011). Plots were designed in R. Annual means of hybridization rates in 250 and 300 m depths were compared with Wilcoxon rank sum test. Hybridization rates of Thaumarchaea were checked for analysis of variance (one-way ANOVA). The* post hoc* Tukey Honestly Significant Difference (HSD) test was applied on all 36 samples from both depths to check for significant differences. The parametric coefficient of variation (%CV) was used to obtain a standardized indication of variability of the data of microbial community composition obtained with CARD-FISH analysis. The sampling variability of the hybridization rate of Thaumarchaea was calculated as the mean CV from all samples.

## 3. Results

### 3.1. Lake Maggiore Chemical and Physical Parameters

**Figure 1:**
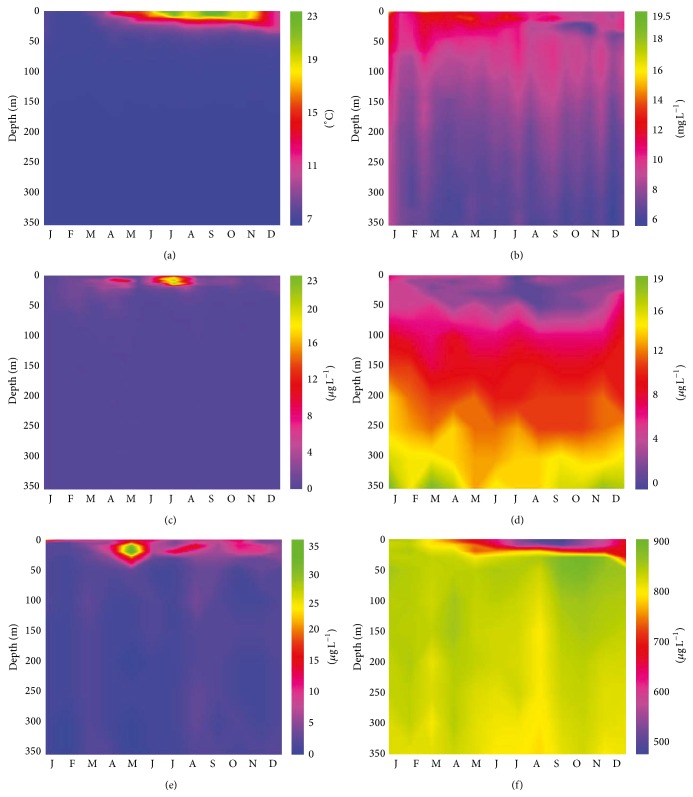
Intra-annual vertical profiles of physical and chemical parameters at the Ghiffa pelagic station during 2011 (45°57′N, 3°46′W: 370 m depth). Temperature in °C (a), oxygen concentrations in mg L^−1^ (b), and chlorophyll *a* concentrations in *μ*g L^−1^ (c) were measured every two weeks at intervals of 0.5 m. Concentrations of reactive phosphorous in *μ*g L^−1^ (d), ammonium in *μ*g L^−1^ (e), and nitrate in *μ*g L^−1^ (f) were measured at 0, 5, 10, 20, 30, 50, 10, 150, 200, 250, 300, and 350 m depth.

Water temperature in Lake Maggiore was approximately 6.5°C in the whole water column during winter 2011. From March on, the temperature of the lake water column down to about 50 m depth increased to a maximum of 23°C at the surface in July and September (Figure 1(a)). The level of dissolved oxygen in the epilimnion varied throughout the year, with a maximum of 19.5 *μ*g L^−1^ at the end of April, concomitant with the onset of temperature increase and a detected chlorophyll* a* (Chl* a*) maximum. In waters below 150 m depth, the oxygen concentration was constant at 6–8 *μ*g L^−1^, indicating a full oxygenation of the deeper layers of the lake during the whole year (Figure 1(b)). Maxima of Chl* a* concentrations were detected between 2 and 10 m depths in April (13 *μ*g L^−1^) and July (23 *μ*g L^−1^) corresponding to spring and summer phytoplankton blooms. Below 20 m depth, Chl* a* concentrations constantly ranged between 0 and 2 *μ*g L^−1^ (Figure 1(c)). Reactive phosphorus (RP) increased with depth and was depleted in the epilimnion between March and October. RP concentrations of 12–14 *μ*g L^−1^ at 200 m depth and up to 19 *μ*g L^−1^ at 350 m remained constant throughout the whole year (Figure 1(d)). Ammonium (N-NH_4_^+^) concentrations were close to instruments detection limits below 50 m depth (0–5 *μ*g L^−1^) without any seasonal change, while there was a local maximum at 10 m depth at the beginning of May (Figure 1(e)). Nitrite (N-NO_2_) was always below the detection limit through the whole water column. Nitrate (N-NO_3_) had contrasting trends below 50 m, while in the epilimnion it decreased in summer. Mean annual nitrate concentrations at 200 and 350 m depths were around 830 *μ*g L^−1^ (Figure 1(f)).

Dissolved inorganic carbon (DIC, Figure S1) increased with depth (13.19 ± 0.48 mg L^−1^ at 50 m, 13.67 ± 0.32 mg L^−1^ at 200 m, and 14.09 ± 0.37 mg L^−1^ at 350 m); limited but significant variations (1-way ANOVA, *p* < 0.001) were detected over the year (±0.5 mg L^−1^). The measured annual means of total organic carbon (TOC, Figure S1) were 1.0 (±0.26) mg L^−1^ at 50 m depth, 0.88 (±0.24) mg L^−1^ at 200 m, and 0.83 (±0.28) mg L^−1^ at 350 m. Mean TOC value for River Maggia was 1.13 (±0.44) mg L^−1^, comparable to the other main inflows, that is, 0.39 ± 0.88 mg L^−1^ for River Ticino and 0.91 ± 0.52 mg L^−1^ for River Toce.

### 3.2. Microbial Abundance and Community Composition

**Figure 2:**
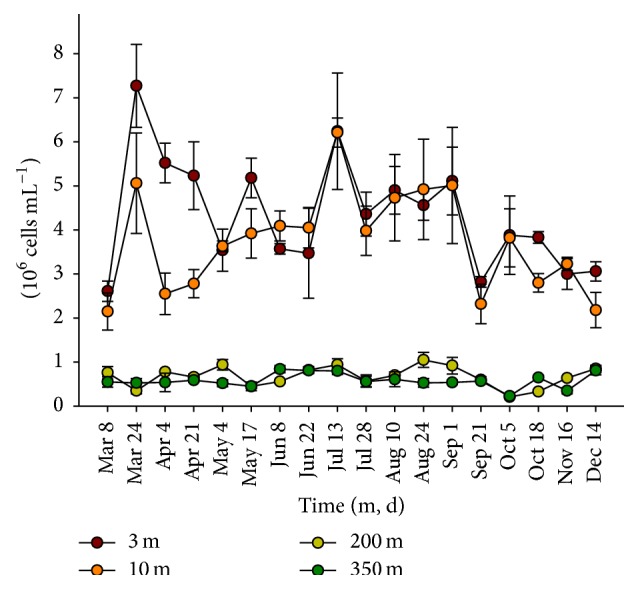
Total prokaryotic abundance (±s.d.) at 3, 10, 200, and 350 m depth at Ghiffa pelagic station, Lake Maggiore, in 2011 obtained from counts of DAPI stained cells.

Total abundance of prokaryotic cells (Bacteria and Archaea) displayed seasonal maxima of up to 7.3 × 10^6^ cells mL^−1^ at 3 and 10 m depth concomitant with the spring (March-April) and summer (July) phytoplankton blooms. No seasonal trend was visible in the deep water layers (200 m and 350 m depth) where the overall abundance was generally lower than 1 × 10^6^ cells mL^−1^ (Figure 2).

**Figure 3:**
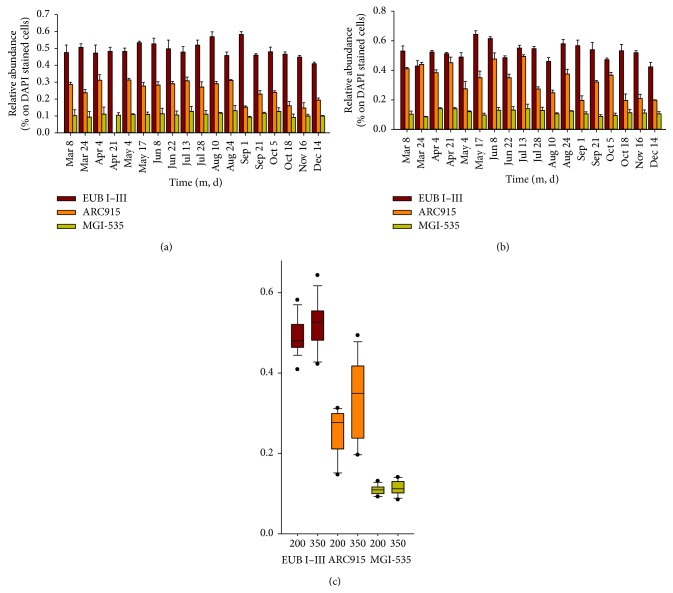
Relative proportions (±s.d.) of hybridized cells in Lake Maggiore in 2011 (Ghiffa sampling station) for Bacteria (probe EUB I–III), Archaea (probe ARC915), and Thaumarchaea of the Marine Group I (probe MGI-535) at 200 m (a) and 350 m (b). Boxplot of hybridization rates (c) for the same samples (Ghiffa station, 2011). Whiskers indicate Tukey's 1.5 IQR. Significant differences in mean between 200 m and 350 m were detected for ARC915 (*p* < 0.01) and EUB I–III (*p* = 0.05).

Total hybridization rates (sum of probes ARC915 and EUB I–III) ranged between 60 and 99% of DAPI stained cells except for two outliers (June 8, 109.1%; July 13, 104.5%). The proportion of hybridized Bacteria (probe EUB I–III) ranged from 40.9% to 58.2% of total DAPI stained cells (mean: 49.1 ± 4.3%, Figure 3) at 200 m and from 42.3% to 64.3% (mean: 52.3 ± 5.8%) at 350 m. Relative abundance of Archaea (probe ARC915) ranged from 14.7% to 31.3% (mean: 25.3 ± 5.8%) at 200 m and from 19.6% to 49.4% (mean: 33.4 ± 10.0%) at 350 m depth. The newly designed probe MGI-535 targets 2108 almost full-length sequences affiliated with MGI-Thaumarchaea (91.1% clade coverage). By allowing one mismatch, this probe theoretically detects 68 additional sequences affiliated with the SAGMCG-1 clade; however, all false-positive hits have a strong central mismatch. Thus, this probe is very specific for MGI-Thaumarchaea. Relative abundance of MGI-Thaumarchaea ranged from 9.2% to 13.1% (mean: 10.9 ± 1.2%) at 200 m and from 8.5% to 14.1% (mean: 11.5 ± 1.2%) at 350 m. All three groups showed no significant variation in relative proportions throughout the sampling period, neither at 200 m nor at 350 m depth (Figure 3). The two depths significantly differed for ARC915 (*p* < 0.01) and EUB I–III (*p* = 0.05), while no significant difference was detected for MGI-535 (Wilcoxon rank sum test). Seasonal variability (coefficient of variation, CV) was higher for ARC915 (22.88% at 200 m, 29.9% at 350 m) than for EUB I–III (8.7% at 200 m, 11.2% at 350 m) and MGI-Thaumarchaea (10.8% at 200 m, 15.6% at 350 m) where sampling variability was within the range of the overall variability (Figure S2). Hybridization rates of MGI-Thaumarchaea were tested by ANOVA to confirm the presence of true difference between the samples (*p* = 0.014 at 200 m, *p* < 0.01 at 350 m) and a Tukey HSD* post hoc* test revealed that differences between samples were higher at 350 m than at 200 m depth. Total numbers of MGI-Thaumarchaea were 7.6 × 10^4^  ± 2.5 × 10^4^ cells mL^−1^ at 200 m and 7.3 × 10^4^  ± 1.9 × 10^4^ cells mL^−1^ at 350 m depth. There was no significant difference between the two depths and there were no significant intra-annual variations.

### 3.3. Analysis of Ammonia-Oxidizing Prokaryotic Communities

**Figure 4:**
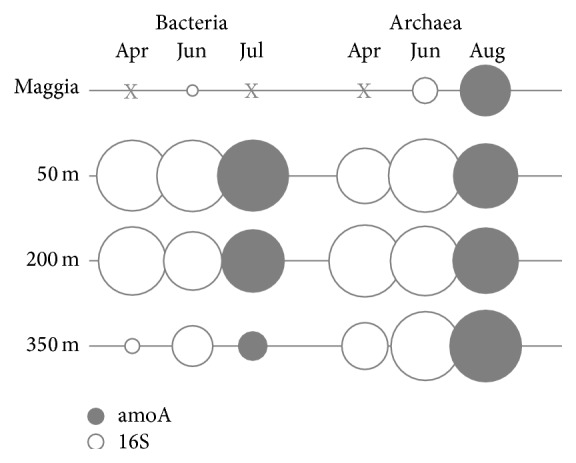
Bubble chart showing relative DGGE band intensities of lake water samples at 50, 200, and 350 m depth (Ghiffa pelagic station) and River Maggia in April, June, and August 2011. 16S rRNA and amoA gene patterns are displayed both for Bacteria and Archaea. X denotes absence of amplified bands. The primer sets applied are different between different amplifications, and the comparison between intensities should be considered only within the same set (vertical columns).

The spatial distribution of ammonia-oxidizing prokaryotes assessed by PCR-DGGE was different for Bacteria and Archaea. Ammonia-oxidizing Bacteria (AOB) were detected at 50, 200, and 350 m depth in the lake but not in River Maggia (Figure 4). No PCR inhibitors were present in river sample based on positive amplification of lake and river DNA mixture. DGGE profiles of the AOB-16S rRNA obtained with CTO-primer set in July consisted of 4 bands and were identical for all depths with a significant decrease in signal intensities from 50 m to 350 m depth. Same pattern of band signal decrease was observed for bacterial* amo*A gene profile in April and June, which consisted of 5 bands. AOA, in contrast, were detected both in the lake and in the river samples except for April when no AOA were detected in the river. The archaeal 16S rRNA DGGE profiles consisted of 4 bands and were identical at all depths in the lake and also in the river samples. The archaeal* amo*A DGGE profiles assessed in April and June consisted of 3 bands. Bands from archaeal 16S rRNA and amoA profiles were excised, reamplified, and separated on denaturing gels till obtaining pure bands for sequencing. The procedure revealed that only one major band of 16S rRNA gene was present in all samples, the other bands being heteroduplexes [43]; sequence LM_50 m_band1 (232 bp) was deposited to GenBank as example (XXXXXXXX). Based on BLAST analysis, sequences obtained from samples at 50-200-350 m depth and River Maggia showed between 98 and 100% identity with the partial 16S rRNA gene sequences of the uncultured Thaumarchaeota clone VWS114 detected by Vissers et al. [15] in Lake Lucerne. Excision and reamplification of bands from AOA_amoA gel profile revealed also that only one major band was present, the others being heteroduplexes; sequence LM_200 m_band5 was deposited to GenBank as example (accession number XXXXXXXX); BLAST analysis showed 99% identity with isolated DGGE gel bands of uncultured crenarchaeon from marine environment and isolated clones from estuarine environments.

### 3.4. Phylogenetic Diversity of Thaumarchaea

Three clone libraries specific for thaumarchaeal 16S rRNA were constructed from lake samples from spring (March) and autumn (September) and from a River Maggia sample from summer (August). A total number of 75 clones were sequenced and phylogenetically analyzed (approximately 900 bp length), whereof 21 and 22 originated from Lake Maggiore from spring and autumn, respectively, and 32 from River Maggia. Our newly designed reverse primer (Table 2) is specific for Thaumarchaeota (79% coverage) and excludes Bacteria even with allowing unspecific PCR conditions.

**Figure 5:**
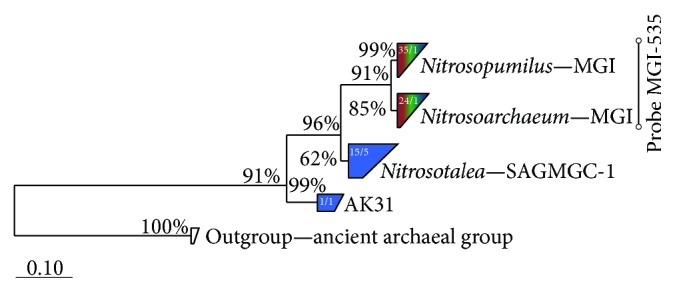
Bootstrapped maximum likelihood tree of sequenced 16S rRNA genes (GTR-GAMMA method, 1000 iterations). Blue colour indicates clones derived from River Maggia, while green and brown indicate clones from Lake Maggiore from spring (March) and autumn (September). Clusters in brown, green, and blue contain sequences from all three clone libraries. Numbers inside clusters refer to the number of sequences and OTUs, respectively. The bar at the bottom applies to 10% sequence divergence. The* Nitrosopumilus* (MGI) cluster contains sequences of* N. maritimus* (CP000866), “*Ca*.* N. koreensis*” (CP003842), and “*Ca*.* N. salaria*” (AEXL02000090); the cluster* Nitrosoarchaeum* contains sequences of “*Ca*.* N. koreensis*” (AFPU01000001) and “*Ca*.* N. limnia*” (AHJG01000224). The other two clusters include sequences of uncultured Archaea (reference sequences are HE589644 and KC437195 for SAGMGC-1 and AK31, resp.). For more details, see Figure S3 in Supplementary Material available online at http://dx.doi.org/10.1155/2015/590434.

The 75 sequences were grouped in 8 OTUs on the base of ≥98% sequence similarity (Figure 5) and were all associated with Thaumarchaea. The River Maggia sample showed the highest diversity with 8 OTUs and a Shannon index of 0.75, whereas only two OTUs were recovered from Lake Maggiore samples and the diversity indices were 0.13 and 0.30 for spring and autumn, respectively. Rarefaction analyses suggest that the diversity of thaumarchaeal 16S rRNA genes was almost fully covered in Lake Maggiore clone libraries, whereas the River Maggia was still undersampled (Figure S4).

Phylogenetic analyses revealed that the two OTUs shared by all 3 libraries belonged to the Marine Group I (MGI) (Figure 5, Figure S3), whereof one OTU was affiliated with the genera* Nitrosopumilus* (35 sequences) and* Nitrosoarchaeum* (24 sequences). These two OTUs represented the majority of all obtained sequences (47% and 32%, resp.) with most of them deriving from the Lake Maggiore clone libraries. Sequences of OTU1, affiliated with* Nitrosopumilus* sp., were closely related to other uncultured thaumarchaeal sequences gained from Lake Lucerne (Figure S3). Half of the sequences of OTU2 (affiliated with* Nitrosoarchaeum* sp.) originated from the River Maggia library (12 sequences) and the closest relatives also derived from either lakes, rivers, or groundwaters. The six remaining OTUs were exclusively gained from the River Maggia and grouped with the South African Gold Mine Group (SAGMGC-1, genus* Nitrosotalea*, 15 sequences, OTUs 3–7) and the AK31 clade (1 sequence, OTU8). The closest relatives of OTUs 3, 4, and 8 were gained from rivers or springs, while OTUs 5–7 were closely related to sequences originating from different lakes (Figure S3).

## 4. Discussion

In recent years, particular emphasis has been put on the resident microbial community of the hypolimnion of deep lakes. Lake Maggiore is one of the best studied deep oligotrophic lakes (maximum depth 370 m) and represents an ideal model for this type of lakes. Increasing proportions of Archaea with depth have been reported for Lake Maggiore [18] and other deep lakes [14, 15]. Archaea proved to contribute significantly to the carbon cycle and were responsible for 28% of total dark [^14^C]-HCO_3_ uptake in Lake Maggiore [44]. Dark CO_2_ fixation rates of 187.7 ± 15 *μ*g C m^−3^ d^−1^ in the deep hypolimnion [44] matched those observed for marine ecosystems [45]. Thus, these microbes seem to play a similarly important role in the carbon cycle as their marine counterparts. In this study, we showed that the hypolimnetic thaumarchaeal population of Lake Maggiore accounted for about 11% of the whole prokaryotic community, with no significant intra-annual variation, as demonstrated by the CV values of CARD-FISH hybridized cells (Figure 3, Figure S2). This is in contrast to a study by Callieri et al. 2009 [18], where a peak up to 40% Thaumarchaea was detected during summer 2007. This discrepancy could either be caused by variations in the climatic conditions of the two years or could be related to the different probes used for CARD-FISH hybridization for Thaumarchaea in the two studies. The probe MGI-535 was specifically designed for this study and it derived from the traditional probe CREN537 [45] used by Callieri et al. [18]. The new probe showed brighter fluorescence and lower background signals; thus, hybridized cells could be distinguished much better from background signals. Moreover, thaumarchaeal proportions presented here are in line with other aquatic environments, that is, high mountain lakes [14] or the tropic South Pacific Oxygen Minimum zone, where MGI-Thaumarchaea comprised up to 19% of total picoplankton [46].

Regarding the environmental parameters, temperature and oxygen were stable through the year in the hypolimnion (i.e., from 50 to 350 m), with very limited nutrient concentrations. This is typical of deep subalpine lakes where, as for oceans, a huge water mass is buffering the variations in temperature and dissolved oxygen. In the case of Lake Maggiore, in the year 2011 no full overturn of water occurred and the oxygenation of deep water was maintained as always by the riverine inputs [23]. Ammonia and nitrite concentrations were close to or below the detection limit, not allowing any inference with nitrification activities for which more specific and accurate techniques are needed [44, 47]. The absence of patterns in AO abundance and distribution did not allow any correlation with the detected variations in dissolved inorganic carbon (DIC) which is a source of CO_2_ for autotrophic carbon fixation operated by ammonia oxidizers. The stable ad nutrient-limited conditions of the hypolimnion are typically oligotrophic and suitable to harbour mesophilic Thaumarchaea.

Another new aspect of this study is represented by the observation of temporally stable populations of both ammonia-oxidizing Bacteria and Archaea in the deep water layers, from 50 to 350 meters. Significant variations in DGGE band signal intensity showed that AOB were more abundant at 50 m and decreased with depth, while AOA had the opposite pattern reaching their maximum band signal intensity at 200 and 350 m depth. The results presented in this study are necessarily limited to a first observation; however, they represent a first indication of a segregation between AOA and AOB in the hypolimnion of deep large lakes, which can be in line with other studies in different aquatic environments (i.e., sediment lakes [16], estuarine groundwater [48], and high latitude oligotrophic lakes [49]) where quantitative analyses were applied. Despite some conflicting results (see reviews [3, 50]), physiological studies on both bacterial and archaeal isolates suggest that Archaea are better adapted to low ammonia concentrations and higher ability to live at lower oxygen concentrations [51] and might be also more inhibited by UV radiation than Bacteria [10]. Moreover, in our study, we observed the retrieval of AOA and the total absence of AOB in the river water, which fed and oxygenated the deep hypolimnion, suggesting a potential input of Archaea in the lake directly from River Maggia and its catchment area.

The resident Thaumarchaea community of the deep hypolimnion of Lake Maggiore was confirmed as seasonally stable and not very diverse, considering that only two identical OTUs were detected in April and September (Figure 5, Figure S3) and the Shannon diversity indices were 0.13 and 0.30 in April and September, respectively. A low diversity and seasonal persistence of Thaumarchaea was also reported for the hypolimnion of Lake Lucerne, Switzerland [15], and Lakes Annecy and Bourget, France [52]. Not surprisingly, 16S rRNA sequences gained from these lakes clustered together with OTU1 (*Nitrosopumilus*, MGI), the OTU that accounted for the majority of sequences from all three clone libraries (35 sequences, Figure S3). Interestingly, these microbes were not detected in another deep oligotrophic lake (Lake Redon, Spain [14]), while OTU2 (*Nitrosoarchaeum*, 24 sequences from all three clone libraries) grouped together with sequences obtained from this habitat. Therefore, Thaumarchaea of the genera* Nitrosopumilus* and* Nitrosoarchaeum* might be regarded as core groups dwelling in deep zones of freshwater lakes. Both OTUs consisted of sequences from all three clone libraries; however, OTU1 contained more sequences from the spring sample of Lake Maggiore, while OTU2 contained more sequences from River Maggia and the autumn sample of the lake (Figure S3). This might hint at a distinct seasonality of different genotypes of the MGI Thaumarchaea in Lake Maggiore. We could not distinguish between these two genotypes via CARD-FISH as our newly designed probe targets all MGI Thaumarchaea; thus, the observed seasonal stability (Figure 3) might also be caused by a compensatory effect of alternating maxima of different genotypes within the MGI.

The clone library from River Maggia showed the highest number of OTUs (8), and an even higher diversity might be expected (Figure S4). Most OTUs (5) and sequences (16) were affiliated with the SAGMAGC-1 (genus* Nitrosotalea*), a diverse clade originally discovered in borehole waters of South African gold mines [53]. Members of the SAGMAGC-1 closely related to our OTUs have been mainly found in lakes [14, 54], springs, and rivers [55]. Three OTUs consisting of only 1 or 2 sequences (OTUs 5–7, Figure S3) were closely related to lake samples that were gained from the air-water surface microlayer [14, 54]. The overall composition of the archaeal community of River Maggia seems to be composed of either clades of terrestrial or aquatic origin, suggesting the concomitant presence of autochthonous aquatic cells and of cells transported into the river from the soils of the catchment area. A vertical niche segregation of MGI and SAGMGC-1 was described for Lake Redon, with MGI predominantly inhabiting the hypolimnion, while SAGMGC-1 were mainly present in the upper water layers, the air-water surface microlayer, and slush samples [14]. This is in consistency with our results, as we could not detect SAGMGC-1 in our clone libraries from the deep hypolimnion of Lake Maggiore (Figure 5). A high number of sequences gained from River Maggia were also present in the prominent OTUs 1 and 2 (MGI, Figure 5, Figure S3). The river discharges its water directly in the hypolimnion of the lake due to density constraints [23]. Thus it is likely that the river, which collects water from a large catchment basin and harbours a very diverse thaumarchaeal community, serves as inoculum for the deep hypolimnion of the lake. Environmental filtering selects microbes that are best adapted to a new environment, as was reported for frequently flooded rock or cave pools [56, 57]. This kind of species sorting might also occur in Lake Maggiore, as the seasonally very stable conditions in the deep hypolimnion seem to favour the prevalence of MGI Thaumarchaea.

Finally, Thaumarchaea represent an important fraction of the overall microbial community of Lake Maggiore. Their presence, limited to the hypolimnion of the lake, is constant through the year. Their low diversity in the lake, in comparison to the tributary River Maggia, allows a speculation on their allochthonous origin, filtered by ecological adaptation in the Lake Maggiore, where competition for resources is high, and specific environmental factors are in action.

## Supplementary Material

Bootstrapped maximum likelihood tree of sequenced 16S rRNA genes (GTR-GAMMA method, 1000 iterations). Accession numbers of sequences retrieved in the study are indicated in brackets. Sequences Colored in blue were gained from clone libraries from River Maggia, while sequences gained from Lake Maggiore are colored in green (March) or brown (September). The bar at the bottom applies to 10% sequence divergence. Brackets on the right side mark different OTUs and genus-like clusters.

## Figures and Tables

**Table 1:** CARD-FISH probes used for the analysis of prokaryotic community composition at 200 and 350 m depth in Lake Maggiore. For each probe, short name, target organisms, sequence, formamide concentration (formamide %), references, and probe accession number on probeBase are indicated. ^(a)^Probe MGI-535 was specifically designed for this study, targeting Marine Group I (MGI) Thaumarchaea.

Probe name	Target organism	Sequence (5′-3′)	Formamide %	Reference	Acc. number
ARC915	Most Archaea	GTG CTC CCC CGC CAA TTC CT	40	Stahl and Amann (1991) [24]	pB-00027
MGI-535^(a)^	Marine Group I Thaumarchaea	TCC TGA CCA CTT GAG GTG CTG G	45	This study	
EUB-I-III	Most Bacteria	GCW GCC WCC CGT AGG WGT	55	Daims et al. (1999) [25]	pB-00159-61

**Table 2:** Primer sets and conditions used for 16S rRNA and AmoA genes amplification and cloning of ammonia-oxidizing prokaryotes. An initial denaturation (94°C for 5 min) and final elongation (72°C for 20 min) steps were performed to all reactions. ^(a)^Touchdown 0.5°C per cycle, from at 68°C to 60°C; ^(b)^+1 sec per cycle. Final elongation = 5 minutes. ^(b)^New: reverse primer, specifically targeting Thaumarchaeota (thaum922r: 5′-TTG TGG TGC TCC CCC GCC-3′). f: forward; r: reverse primer; ^(c)^primers contained 40-nucleotide-long GC-sequence at the 5′ end for Denaturing Gradient Gel Electrophoresis [31].

Target gene/group	Primer pair	Cycles	Denaturation		Annealing		Elongation		Reference
			°C	s	°C	s	°C	s	
Archaea 16S rRNA	arc344f^(c)^ arc915r	30	94	60	68^(a)^	60	72	90	Stahl and Amann (1991) [24]

Thaumarchaea 16S rRNA	arc21f thaum922r^(b)^	30	94	60	62	60	72	80	DeLong (1992) [32] This study

AOB 16S rRNA	CTOf-mix^(c)^ CTOr	35	92	30	57	60	72	45^(b)^	Kowalchuk et al. (1997) [33]

AOB *amoA *	*amoA*-1f^(c)^ *amoA*-2r	35	94	60	60	90	72	90	Rotthauwe et al. (1997) [34]

AOA *amoA *	Arch-*amoA*F^(c)^ Arch-*amoA*R	30	94	45	53	60	72	60	Francis et al. (2005) [35]
