# Supplementary material for: Ecology and Distribution of Thaumarchaea in the Deep Hypolimnion of Lake Maggiore
Source: Archaea. 2015 Aug 25;2015:590434. doi: 10.1155/2015/590434 (PMC4561949; doi:10.1155/2015/590434)

Supplementary Information

Fig. S1: Dissolved Inorganic Carbon (DIC, left panel) and Total Organic Carbon (TOC, right panel) in Lake Maggiore (Ghiffa pelagic station) at 50, 200, 350 m, and in River Maggia (TOC only). Data represent the mean of 18 sampling dates throughout 2011.

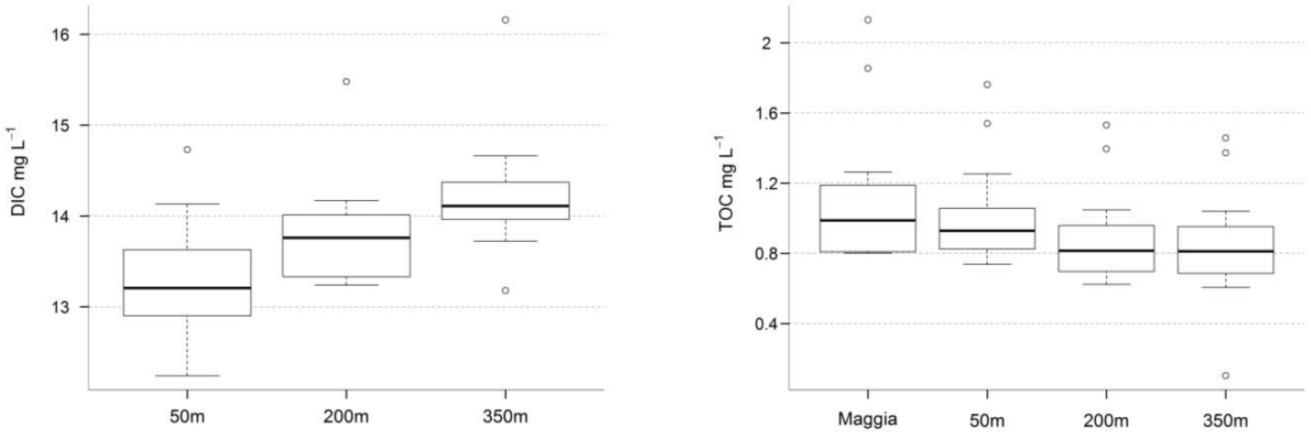

Fig S2: All year data Coefficient of Variation (CV) of cells hybridized either with probes MGI-535, ARC915, or EUB I-III at 200 and 350 m (Ghiffa station), and CV of intrinsic sampling variability for MGI-535.

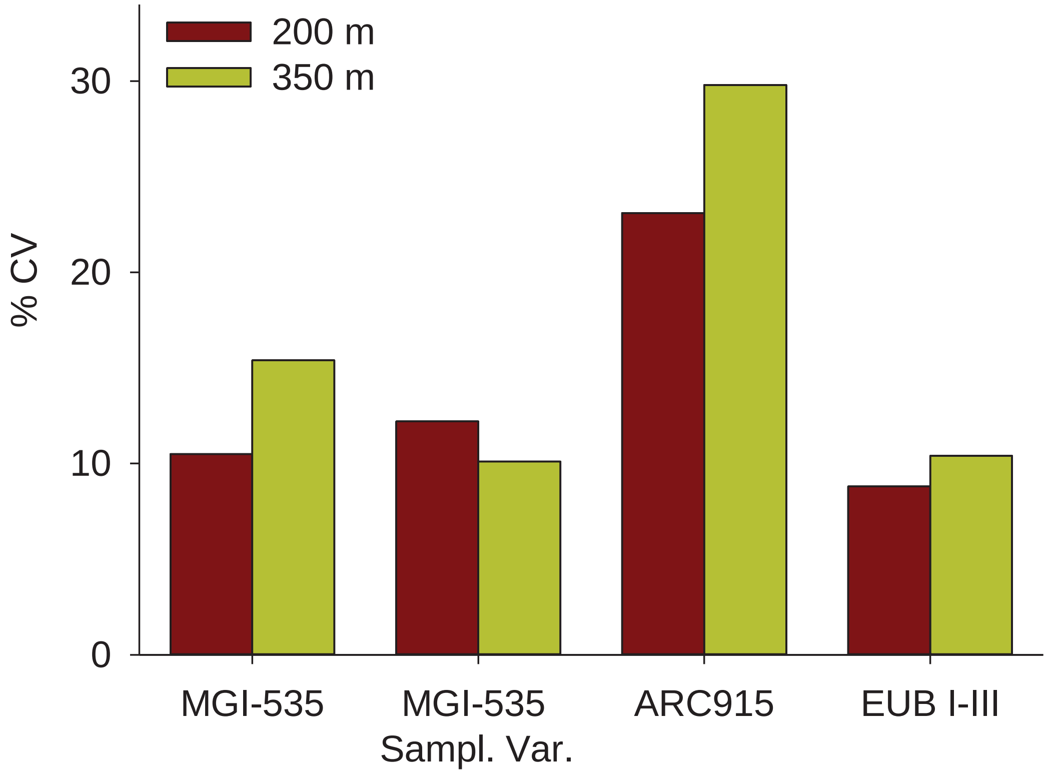

Fig. S3: Bootstrapped maximum likelihood tree of sequenced 16S rRNA genes (GTR-GAMMA method, 1000 iterations). Accession numbers of sequences retrieved in the study are indicated in brackets. Sequences colored in blue were gained from clone libraries from River Maggia, and sequences gained from Lake Maggiore are colored in green (March) or brown (September). The bar at the bottom applies to 10% sequence divergence. Brackets on the right side mark different OTUs and genus-like clusters.

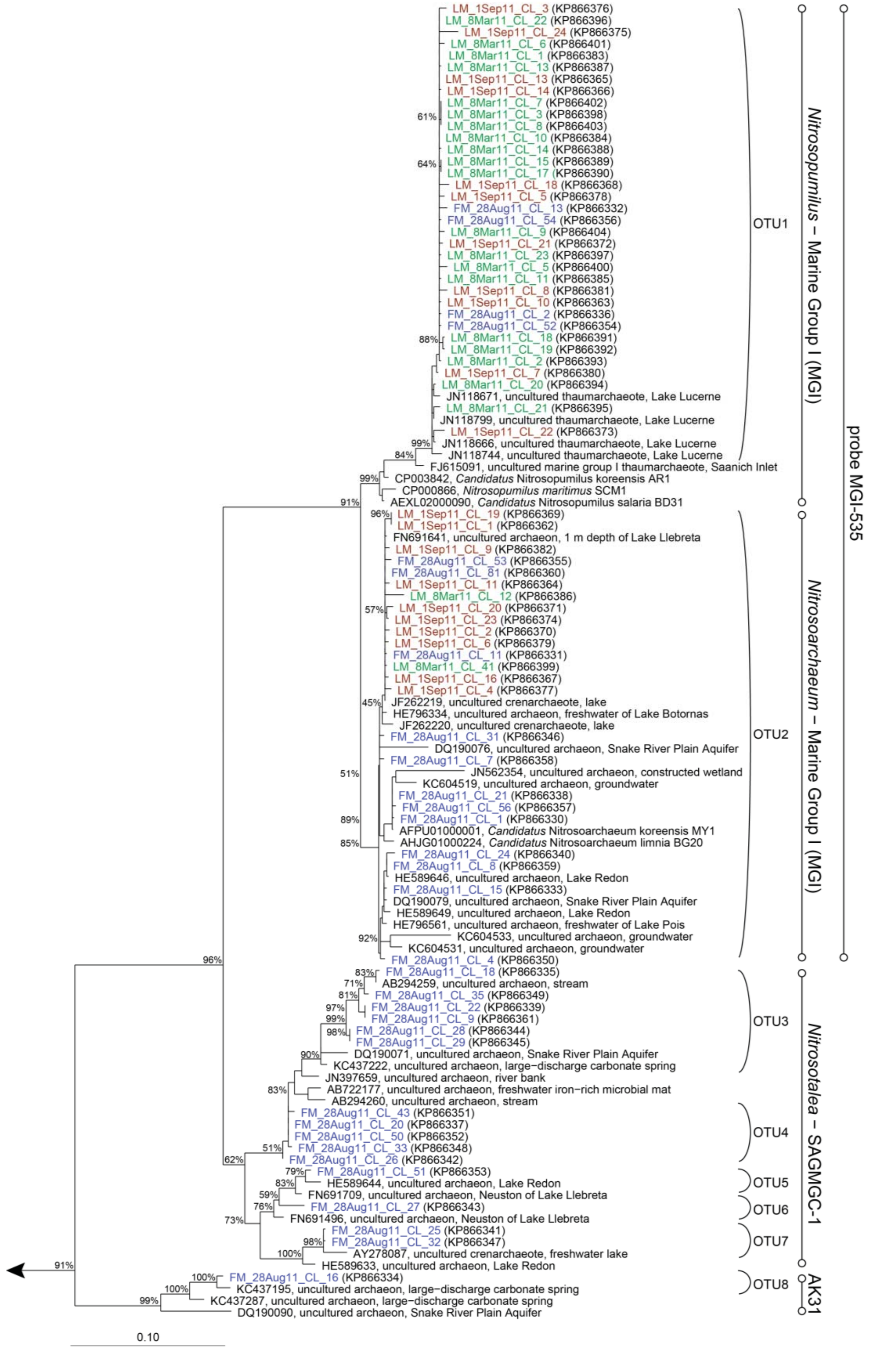

Fig. S4: Rarefaction curves (Sobs Mao Tau) and confidence interval (95%) indicating archaeal 16S rRNA gene richness within clone libraries derived from Lake Maggiore 350 m in spring and fall, and River Maggia in fall. OTUs were clustered at a similarity level of 98%.

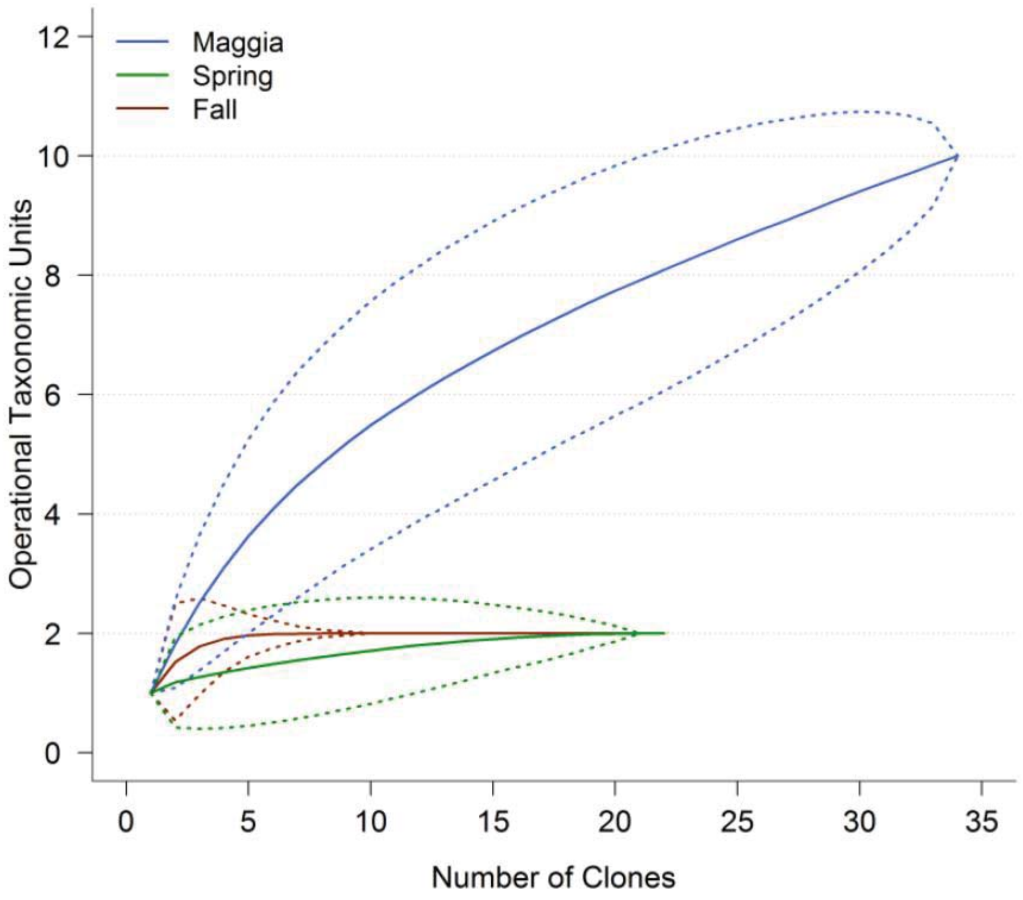

Supplement: Supplementary file 1 — Bootstrapped maximum likelihood tree of sequenced 16S rRNA genes (GTR-GAMMA method, 1000 iterations). Accession numbers of sequences retrieved in the study are indicated in brackets. Sequences Colored in blue were gained from clone libraries from River Maggia, while sequences gained from Lake Maggiore are colored in green (March) or brown (September). The bar at the bottom applies to 10% sequence divergence. Brackets on the right side mark different OTUs and genus-like clusters. [file 590434.f1.pdf]
